# 90-day mortality of severe sepsis and septic shock is reduced by initiation of oral beta-blocker therapy and increased by discontinuation of a pre-existing beta-blocker treatment

**DOI:** 10.1186/2197-425X-3-S1-A88

**Published:** 2015-10-01

**Authors:** C Fuchs, C Scheer, S Wauschkuhn, M Vollmer, S Rehberg, K Meissner, SO Kuhn, S Friesecke, P Abel, M Gründling

**Affiliations:** Klinik für Anästhesiologie, Universitätsmedizin Greifswald, Greifswald, Germany; Klinik für Innere Medizin B, Universitätsmedizin Greifswald, Greifswald, Germany

## Introduction

A continuous infusion of the beta-blocker esmolol has recently been demonstrated to increase stroke volume without increasing norepinephrine dosage or impairing microcirculation in patients with septic shock [1]. In addition, a pre-existing oral therapy with beta-blockers was associated with a potential 28-day mortality survival benefit in septic patients [2]. The influence of a new initiated or discontinued oral beta-blocker therapy upon 90-day mortality, however, remains unclear.

## Objectives

We hypothesized that a new initiated therapy with oral beta-blocker reduces 90-day mortality in patients with severe sepsis or septic shock, while discontinuation of a pre-existing treatment increases it.

## Methods

The present observational, single-center cohort study of intensive care unit (ICU) patients with primary severe sepsis or septic shock at the University Hospital of Greifswald, Germany, was performed from January 2010 to December 2013. The local ethics committee approved the study (Identifier: BB 133/10) and waived a written informed consent because of the anonymous data collection and the quality saving and observing character of the study. The study complied with the Declaration of Helsinki and was performed according to the responsible data protection board. The oral administration of beta-blockers (bisoprolol, carvedilol, metoprolol, nebivolol, atenolol, talinolol, propranolol, sotalol) before as well as during ICU stay and 90-day mortality were registered. Categorical data are expressed as percentages and counts.

## Results

580 adult patients were included.

Discontinuation of a pre-existing administration with beta-blockers during sepsis therapy was associated with an increased 90-day mortality of 71% (42/59) compared to 42% (96/226) in patients with a continued therapy (OR 3.35; 95% CI 1.80 - 6.23) (p < 0.001). The number needed to harm was 3.48 (95% CI 2.38 - 6.46).

In contrast, a new initiated therapy of oral beta-blockers reduced 90-day mortality from 42% (77/181) in patients without beta-blocker therapy before and during sepsis to 28% (32/114) (OR 0.52, 95% CI 0.32 - 0.87) (p < 0.05). The number needed to treat was 6.9 (95% CI 3.93 - 28.40).

A chronic, pre-existing beta-blocker therapy did not improve 90-day mortality if continued during sepsis therapy in comparison to patients who did neither get beta-blocker treatment before or during sepsis (OR 1.00; 95% CI 0.68 - 1.49) (p > 0.05).

## Conclusions

The present results suggest that a pre-existing therapy with oral beta-blockers should not be stopped in patients with severe sepsis and septic shock. In addition, the new initiation of beta-blocker therapy should be considered in these patients after initial stabilization. Randomized, controlled studies are required to confirm these results.
